# AIDS-Related EBV-Associated Smooth Muscle Tumors: A Review of 64 Published Cases

**DOI:** 10.4061/2011/561548

**Published:** 2011-03-10

**Authors:** Bibianna Purgina, Uma N. M. Rao, Markku Miettinen, Liron Pantanowitz

**Affiliations:** ^1^Department of Pathology, Presbyterian-Shadyside Hospital, University of Pittsburgh Medical Center, Pittsburgh, PA 15232, USA; ^2^Department of Soft Tissue and Orthopedic Pathology, Armed Forces Institute of Pathology, Washington DC 20306-6000, USA

## Abstract

The number of reported cases of smooth muscle tumor (SMT) arising in patients with AIDS has been increasing since the mid-1990s. The aim of this study is to characterize the epidemiology, clinical manifestations, pathologic features, prognosis and, management of Epstein-Barr virus-related SMT (EBV-SMT) in patients with AIDS. An English language literature search identified 53 articles including 64 reported cases of EBV-SMT. The majority of these reports involved patients who were young, severely immunosuppressed, and had multifocal tumors. The central nervous system was the most common site to be involved. Histologically, tumors had smooth muscle features and were immunoreactive for muscle markers and all but two tumors demonstrated the presence of EBV by either immunohistochemistry, in situ hybridization, and/or PCR. While mitoses and/or necrosis were used to separate leiomyoma from leiomyosarcoma, these features did not correlate with clinical outcome. Treatment included primarily resection, and less often radiotherapy, chemotherapy and highly active antiretroviral therapy (HAART). Overall, EBV-SMTs appear to have variable aggressiveness and clinical outcome and may exhibit a more favorable prognosis compared to conventional leiomyosarcoma. Tumor-related death from EBV-SMT occurred in only 4 of 51 patients.

## 1. Introduction

An increased rate of both acquired immune deficiency syndrome (AIDS) defining cancers (e.g., Kaposi sarcoma, B-cell non-Hodgkin lymphoma, uterine cervical neoplasia) and non-AIDS defining cancers (NADC) like Hodgkin lymphoma, anal cancer, and lung cancer is a well established phenomenon in patients with AIDS. As the human immunodeficiency virus (HIV) pandemic persists and more people are living with chronic HIV infection, the rate and spectrum of NADC continue to grow. There are increasing numbers of case reports and small case series in the literature documenting the occurrence of smooth muscle tumors (SMT) in the adult and pediatric population suffering from AIDS. In fact, SMTs including malignant leiomyosarcoma, prior to the AIDS epidemic, were exceedingly rare in the pediatric population. Now, SMTs appear to be the second most common type of neoplasm arising in children with AIDS [1–3]. EBV-SMT also occurs in posttransplant immunosuppressed patients and individuals with other causes of immunosuppression such as autoimmune disease and common variable immunodeficiency syndrome [4, 5].

Coinfection with Epstein-Barr Virus (EBV) appears to be a necessary cofactor for the development of these tumors. Hence, these tumors have aptly been termed EBV-associated SMT (or EBV-SMT). The association of EBV and SMT in immunocompromised patients was first reported in the early 1990s. Since then there have been many case reports of AIDS-related EBV-SMT ranging from leiomyoma to leiomyosarcoma (LMS). The pathogenesis of EBV-SMT is related to the infection and neoplastic transformation of smooth muscle cells by EBV with clonal expansion. However, the exact mechanism of tumorigenesis is still unclear. It has been reported that these tumors tend to be multifocal with the propensity to arise in virtually any anatomical location. The clinicopathological features of these unique myogenic neoplasms in relation to their biological behavior and management remains to be fully elucidated. To date, there has been no comprehensive evaluation of the published literature on EBV-SMT arising in persons with AIDS. Therefore, the aim of this paper is to review the literature describing SMT occurring in patients with HIV/AIDS in order to characterize their epidemiology, clinical manifestations, pathological features, prognosis, and management.

## 2. Methods

A literature review was performed using PubMed as well as cited references within previously published articles and textbooks for all published cases of SMT in patients with documented HIV infection published in the English language literature. An attempt was made to avoid duplicate cases published in the literature. Data accrued from patients with HIV/AIDS with at least one SMT included publication date and the authors' country of origin, patient demographics (age, gender), HIV details such as mechanism of HIV acquisition, CD4 cell count (cells/*μ*L), HIV plasma RNA level (copies/mL), time to detection/manifestation of SMT from HIV diagnosis (months), tumor type (leiomyoma, leiomyosarcoma or LMS, other SMT subtype), SMT clinical and gross pathology findings (anatomical site, number of tumors present, size in cm), histopathology (number of mitoses/10 high power field or HPF and presence of necrosis), immunohistochemical findings, EBV status by EBV-encoded RNA (EBER) or PCR, therapeutic modality and outcome (recorded as alive with no evidence of disease, alive with disease, dead of disease, dead of another cause, or lost to follow up; measured in months). The data was tabulated and analyzed using descriptive statistics.

## 3. Results

A total of 53 articles were retrieved [1–53], that reported details on 64 confirmed HIV positive patients with SMTs. Although EBER was negative in two SMT cases [9, 19] and EBV testing of the tumors were not performed in five patients [8, 37, 42], these HIV-related SMT were included in this paper. Also included was one case of an EBV-positive myopericytoma [35]. The publications on SMT in HIV-infected individuals spanned a 20-year period (1990–2010), with an equal number being published before and after the year 2000.

### 3.1. Pathogenesis

EBV is a member of the gamma subfamily of herpesvirus. The EBV genome has more than 100 genes, but only a few are relevant in transmission and replication, including the latent membrane proteins (LMP1, LMP2A, and LMP2b), EBV nuclear antigens (EBNA1, EBNA2, EBNA3A, EBNA3B and EBNA3C), and EBV early RNAs (EBER1, and EBER2). EBV is associated with a number of neoplasms in AIDS patients, including Burkitt and Hodgkin lymphoma.

Primary infection with EBV mainly takes place in the oropharynx where the virus undergoes lytic replication during which viral progeny are released. EBV may also initiate active latency, in which one of three types of restricted gene expression programs are established. Type I latency has limited expression only to EBER and EBNA1 and is the latency program seen in Burkitt lymphoma. The type II latency program also includes the latent membrane proteins and is seen typically in nasopharyngeal carcinoma. Type III latency demonstrates expression of LMPs along with all EBNAs and is seen in posttransplant lymphoproliferative disorders (PTLD). PTLD expresses EBNA-2 which is required for the immortalization of B-cells in culture and is associated with B-cell proliferation. Like PTLD, several cases of EBV-SMT have been shown to express EBNA-2 [14, 20, 39, 40]. LMP was mainly negative in HIV-related SMT. Only one case demonstrated weak positivity with LMP-1 [22]. It may be that LMP expression is below that detected by immunohistochemistry in these tumors. Although there were some inconsistent results in cases tested for EBNA-2 and LMP-1 [7, 19, 20, 22, 27, 30, 45], it would appear that overall EBV-SMT demonstrate Type III latency. 

Pathogenesis appears to be related to infection and transformation of smooth muscle cells by EBV. The EBV receptor CD21 was demonstrated on smooth muscle cells in nine tumors, suggesting that EBV entry may occur via this receptor [2, 28, 40]. Some studies found higher levels of CD21 in tumor cells from EBV-SMT in AIDS patients than tumor cells from non-AIDS related smooth muscle neoplasms and normal muscle [2]. This may represent upregulation of the EBV receptor on smooth muscle cells in patients with AIDS [2, 3, 28, 40]. Although increased CD21 expression has not been universally demonstrated, the possibility of transient expression of this receptor prior to neoplastic transformation cannot be excluded. Interestingly, studies attempting to demonstrate CD21 in posttransplant smooth muscle tumors have been unsuccessful [40]. Another possibility is that infection occurs via fusion of smooth muscle cells with EBV-infected lymphocytes [46, 47].

Having entered a smooth muscle cell, it is unclear how EBV infection causes neoplastic transformation with clonal proliferation. Several studies have demonstrated the clonality of EBV-SMT [2, 26, 40, 48], indicating viral infection of the cell prior to clonal expansion and implicating EBV in tumorigenesis. In patients with multiple EBV-SMT, analysis of clonality demonstrated the presence of different clones in tumors from different locations [2, 26, 28, 40, 48]. This supports the hypothesis that multifocal disease is the result of multiple, independent primary lesions rather than metastases. Furthermore, each lesion is likely a separate infection event. Studies looking at EBV copy numbers in EBV-SMT have found variable results and it has been suggested that this variation is the result of EBV being present in the lytic or latent phase [2, 7, 8]. One could expect that patients with active replicating EBV will have EBV detectable in their serum [18, 22, 25, 32, 33].

### 3.2. Epidemiology Findings

The majority (19 of 35 papers, 54%) were published from the USA (see Table 1). Fewer papers were from Asia (20%) including Thailand, Hong Kong, Singapore, and Taiwan, as well as from Europe (17%), from countries like Spain and France. Rare articles were published by authors from Canada and South America (Argentina, Peru). 

### 3.3. Clinical Features

Patients were on average 25 years of age (range, 2.7 to 49 years). Most (66%) were over 20 years old, only three patients (5%) were between the ages of 10 and 20 years, and the rest (30%) were younger than 10 years of age (see Table 2). These findings are similar to two recent series of EBV-SMT in patients with AIDS that reported a greater proportion of patients over the age of 30 years [18, 26]. In general, smooth muscle neoplasms are rare in children, but as previously highlighted by others and our analysis, SMT are disproportionately represented in children with AIDS [1, 3, 28]. Differences in gender were not borne out by these data. In those under 10 years old, the male : female ratio was approximately 1 : 2. For the entire group, very little difference in gender was noted, with an overall male : female ratio of 1.3 : 1 (see Table 2). In 18 patients (28%) HIV infection was acquired by congenital transmission; in the remainder of the cases HIV was acquired by other means (not specified). Mean CD4 count (missing data in 19 cases) was approximately 60 cells/*μ*L (range 0–330 cells/*μ*L). The mean HIV viral load (data available in only 12 cases) was 203,302 copies/mL (range, <50 to >750,000 copies/mL). In three cases, treatment described the use of antiretroviral therapy.

The CNS was a common location, and patients with these tumors often presented with significant neurologic symptoms [7, 8, 11, 14–17, 20, 24]. EBV-SMT should be included in the clinical differential diagnosis for an intracranial or spinal mass arising in a patient with AIDS, which may be amenable to surgical intervention. Patients with EBV-SMT may present with a variety of symptoms based upon tumor location or they may be detected incidentally during imaging studies or at autopsy. EBV-SMT of the gastrointestinal tract, for example, may present with bleeding, abdominal pain, obstruction and perforation [37, 42–44]. Based on published findings, EBV-SMT has a nodular, centrally ulcerated appearance on endoscopic examination [42]. Those with endobronchial tumors may present with cyanosis, fever, and/or pulmonary infections unresponsive to antibiotics [6, 19, 34]. Endobronchial tumors, if large enough, may be visualized on bronchoscopic examination and surgically ablated.

**Figure 1:**
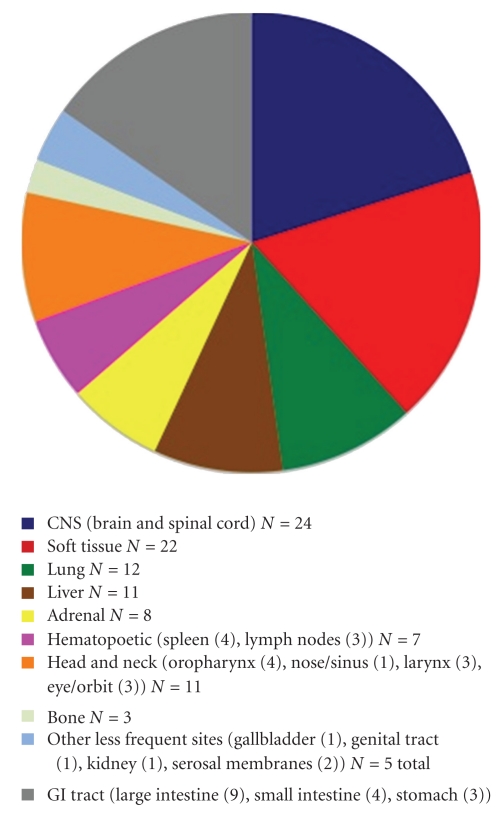
Anatomic location of SMT in HIV-infected patients.

**Figure 2:**
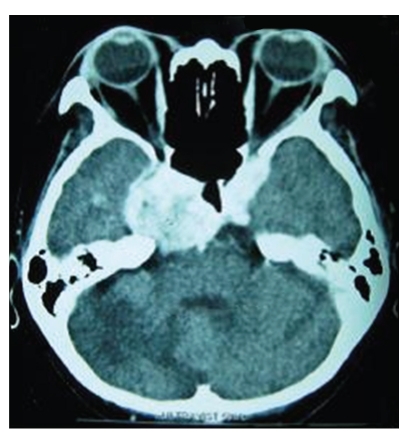
Cranial computed tomography (CT) scan showing a 4 cm enhancing extradural EBV-SMT at the medial aspect of the right tentorium cerebelli, with erosion of the petrous apex and extending into the right optic canal, prepontine cisterns and encasing right carotid (cavernous portion) artery (image reproduced with permission from [13]. The AIDS Reader, UBM Medica).

**Figure 3:**
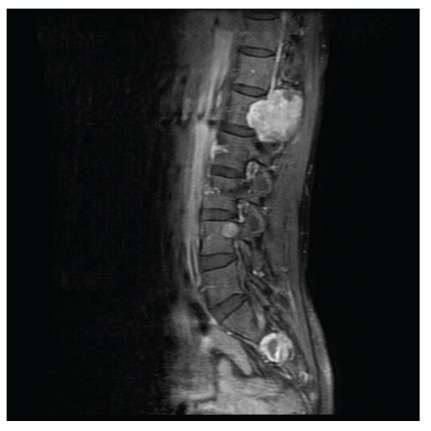
A T1-weighted MRI scan of the spinal cord showing two enhancing extradural hypointense EBV-SMT, approximately 3 cm and 1 cm in diameter, present at the right neural foramina of L3 and S1, respectively (image reproduced with permission from [13]. The AIDS Reader, UBM Medica).

**Figure 4:**
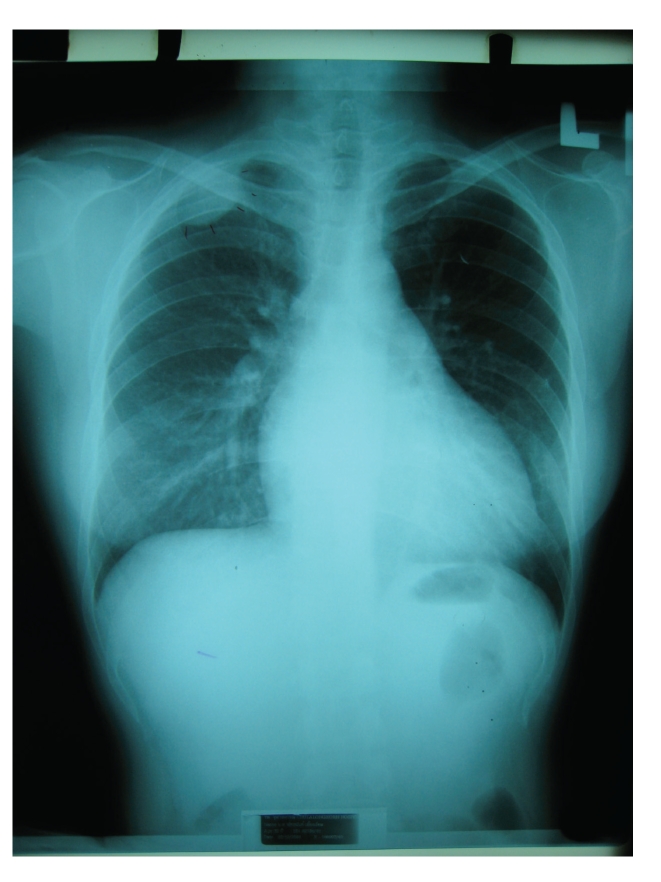
Chest radiography showing an extrapleural EBV-SMT at the apical area of the right lung (image reproduced with permission from [13]. The AIDS Reader, UBM Medica).

**Figure 5:**
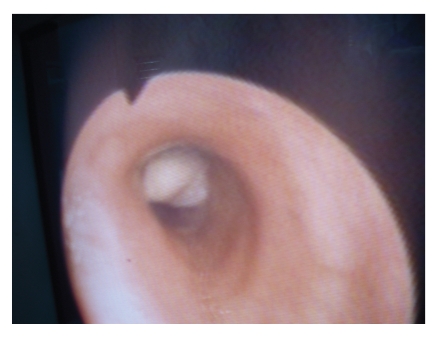
Fiberoptic bronchoscopic examination showing an endobronchial lobulated leiomyoma that obstructed the ostium of the upper lobe in an HIV-positive patient (image courtesy of Dr. Humberto Metta).

In 33 cases, there were multiple SMT, either concurrently or sequentially, and in 20 cases these tumors manifested as solitary lesions. Data on the number of tumors found in patients was unavailable in 12 cases. Multifocal involvement is unusual in smooth muscle neoplasms arising in immunocompetent individuals and is an important feature of EBV-SMT in AIDS patients. The tumor site and frequency (based upon available data in 53 patients) is shown in Figure 1. EBV-SMT involved almost every body location. In cases where there were greater than five tumors per patient, sites of involvement included the brain, lung, liver, adrenals, colon, and soft tissue. In six cases the diagnosis of EBV-SMT was detected only at autopsy. The CNS (brain in 19 cases and spinal cord in 5 cases) is the most common reported location (Figures 2 and 3). Within the CNS, SMT may be dural, epidural, or extradural. Rare cases involved the basal ganglia [11] and pontine cistern [15]. Lung SMT was the second most common tumor reported (Figures 4 and 5). SMT in both peripheral (thigh, gluteal, abdominal wall) and axial (paraspinal) soft tissue sites were reported. All parts of the gastrointestinal tract were involved from the tongue (2 cases), to palatine tonsil (1 case), stomach, small intestine, gallbladder and colon. Bone involvement was documented, mainly of the vertebrae, and in these cases patients had SMT of multiple body sites. Within the larynx, SMT developed in the vocal cord. Genitourinary tract SMT was infrequently reported, including renal involvement in one patient and multiple tumors of the vulva in a 31-year-old female [23]. SMT of the serosal membranes were seen in the pleura and pericardium. The time from HIV diagnosis to SMT presentation ranged from under 1 month to 18 years. There was little difference between tumors that presented within one year and after one year of an HIV diagnosis with respect to the patient age, CD4 count, number of tumors, or patient, outcome.

EBV-SMTs appear to arise when patients exhibit modest immunosuppression due to their underlying HIV infection, given that the mean CD4 count was 60 cells/*μ*L in this study. In only 3 of the 42 cases in which the CD4 levels were available did patients have CD4 cell counts ≥200 cells/*μ*L. Although it is difficult to determine the exact relationship between SMT and HIV viremia based upon data available in only 12 cases, the HIV viral load was on average around 203,302 copies/mL. The time from HIV diagnosis to SMT presentation ranged from under 1 month to 18 years. While more than two thirds of cases were diagnosed within 4 years of an HIV diagnosis, it is likely that chronic HIV infection may play an indirect role. Patients with LMS not only had lower CD4 cell counts, but also more chronic HIV infection.

### 3.4. Pathology Findings

Ten patients were diagnosed with leiomyoma, 25 with LMS, and in 28 cases the SMT was not further characterized, including those cases where tumors were reported to be a SMT of undetermined malignant potential (SMT-UMP). The characteristics of benign and malignant SMT are summarized in Table 3. Leiomyoma was more common in males. Patients with LMS had higher CD4 cell counts and a longer time to presentation. Tumors (in 30 cases with available published data) ranged in size from 0.5 to 14 cm. 

**Figure 6:**
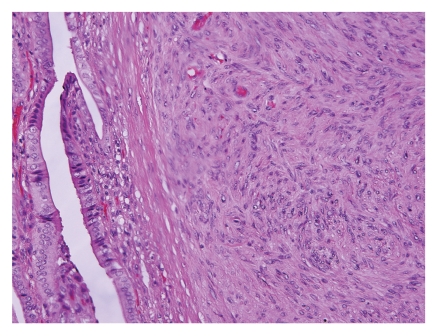
Histopathology of an EBV-associated leiomyosarcomas of the gallbladder is composed of fascicles of mildly atypical spindle cells with blunt-ended nuclei and eosinophilic cytoplasm (H&E stain).

Histologically, EBV-SMT demonstrated interlacing fascicles of mild to moderately pleomorphic cigar-shaped spindle to oval cells with ample eosinophilic cytoplasm (Figure 6). With electron microscopy, EBV-associated smooth muscle neoplasms demonstrated spindle cells with abundant cytoplasmic actin microfilaments with linearly arranged pinocytic vesicles [6, 7, 17, 20]. Some cases described a mild to moderate increase in cellularity and a second population of small round cells with irregular nuclear contours that displayed a smooth muscle phenotype [26, 29, 48]. Other features that have been described include a hemangiopericytic pattern with dilated and branching capillaries and chronic inflammatory cells including intratumoral T lymphocytes [22, 26, 29, 35, 36, 48]. In some cases, tumors appeared to originate from vessel walls, which the authors believed might explain the multiplicity of these tumors [26, 48]. 

Histopathological evaluation of these tumors did not reveal overt features of malignancy in the vast majority of reports. Patients rarely presented with both benign and malignant SMT [12, 28]. Definitive criteria for determining malignancy of smooth muscle neoplasms arising outside the gynecologic, genitourinary and gastrointestinal tracts have yet to be firmly established [54]. It is unclear if published guidelines and recommendations for diagnosing LMS were adhered to in all papers. While tumor size, cellularity, cytologic atypia, necrosis, and hemorrhage may correlate with the malignant behavior of SMTs, the most dependable predictor is the level of mitotic activity [54]. The reported mitotic figure count (34 cases) ranged from 0–19 mitoses/10HPF. In 59% of cases there were 2 or less mitoses/10HPF recorded. Ki67 was performed in three cases and showed a proliferation index from 2-3% (solitary brain LMS) to 12% (vulva LMS) and 18% (SMT of the orbit). Tumor necrosis was an uncommon finding as it was described in only 3 cases. It was noted in two patients with LMS, one in the adrenal gland of a patient that was still alive 20 months later [4], in the colon and rectum of a 7-year-old girl alive with her tumors 36 months later [42], and reported to be only focal in a patient with multiple lung SMT-UMPs [34]. 

### 3.5. Ancillary Study Results

**Figure 7:**
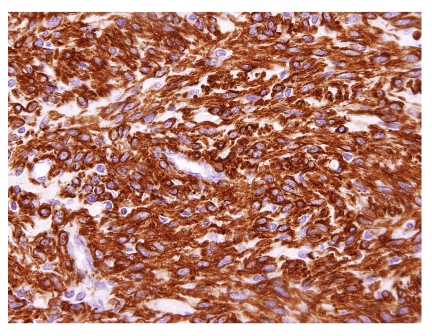
EBV-associated smooth muscle tumor is strongly positive for alpha smooth muscle actin (immunohistochemical stain).

A wide array of immunohistochemical stains were performed in these studies. The results of immunostains showing positive immunoreactivity are shown in Table 4. Most cases showed positive immunoreactivity for smooth muscle markers (Figure 7). In the cases where desmin was reported to be positive, 22 tumors had only rare or focal staining. The use of other muscle markers like caldesmon and calponin were attempted in only rare cases. The immunostains reported to demonstrate negative staining included S100 (27 cases), CD34 (17 cases), EMA (15 cases), HMB45 (11 cases), CD99 (11 cases), Factor VIII (8 cases), cytokeratin (5 cases), c-kit (5 cases), Factor XIII (2 cases), CD31 (2 cases), LNA-1 (2 cases), CD68 (2 cases), GFAP (2 cases), Ulex (1 case), CD23 (1 case), CD10 (1 case), NSE (1 case), ER (1 case), PR (1 case), p53 (1 case), and AFP (1 case). Although immunoreactivity for estrogen and progesterone receptors was only evaluated in one study and reported to be negative in SMT, a hormonal dependent mechanism is likely not involved in the development of these tumors [24]. 

**Figure 8:**
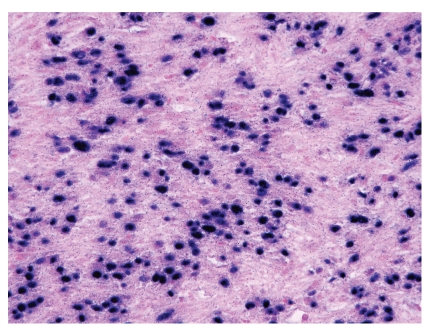
In situ hybridization for Epstein-Barr virus encoded RNA (EBER) shows positive (blue) staining in the tumor cell nuclei.

In studies in which electron microscopy was performed, no nuclear viral particles were detected and the findings were in keeping with a smooth muscle neoplasm [4, 6, 7, 20, 24, 37]. The demonstration of EBV within SMT (Figure 8) was performed in 57 of 63 cases in these published reports and was proven by a positive EBER (96% of tested cases), immunohistochemistry for EBNA (with strong immunoreactivity in 3 of 4 cases) and PCR (3 of 3 cases positive). The presence of high copy numbers of EBV in tumor cells by quantitative PCR was found to be consistent with results of in situ hybridization tests [28]. It has been suggested that the presence of CD21 (EBV receptor) on smooth muscle cells is a putative prerequisite for EBV infection [55], but this is unlikely since the mechanism of EBV entry into different cell types may be by different routes, and it is also uncertain whether the CD21 antigen found on smooth muscle cells is similar to that found on B lymphocytes [56]. Immunohistochemical staining for LMP-1 in addition to EBER was performed in 7 cases [16, 19, 20, 22, 27, 30]. Only one of the cases demonstrated weak immunoreactivity for LMP-1 [22]. One of these cases, an endobronchial leiomyoma arising in a patient with AIDS, was negative for LMP-1 and EBER [19]. The presence of EBV in SMT cells not only supports a role for this herpes virus in the pathogenesis of these tumors, but also serves as a useful diagnostic marker. EBER in situ hybridization should probably be performed on any smooth muscle neoplasm arising in an immunocompromised patient. Classical LMS and leiomyomas arising in immunocompetent patients have not demonstrated an association with EBV [2, 30, 49, 50]. 

### 3.6. Differential Diagnosis

The differential diagnosis of a spindle cell neoplasm arising in the setting of HIV infection includes Kaposi sarcoma and mycobacterial spindle cell pseudotumor. Kaposi's sarcoma is immunoreactive with endothelial cell markers such as CD31 and CD34, and negative for muscle specific antibodies. Kaposi sarcoma lesional cells are also strongly immunoreactive for LNA-1 (antibody directed against Kaposi sarcoma Human Virus), whereas EBV-SMT is not [25–27, 35, 38]. In mycobacterial spindle cell pseudotumor, numerous acid-fast bacilli can be demonstrated within the spindle cells. EBV-SMT did not demonstrate any acid fast bacilli in cases in which a Ziehl-Neelsen stain was performed [15, 22, 37]. Also, unlike SMT, mycobacterial spindle cell lesions are CD68 positive. Other spindle-cell tumors that can be mistaken for EBV-SMT include gastrointestinal stromal tumors (GIST) and follicular dendritic cell (FDC) sarcomas. Although there is some overlap in the immunoprofiles of GIST and EBV-SMT, CD34 and CD117 (c-kit) are specific markers of GIST which were reported to be negative in several cases of EBV-SMT. FDC sarcoma is a rare tumor arising from follicular dendritic cells. Like EBV-SMT, an association with EBV has been demonstrated in these sarcomas. FDC sarcoma is composed of eosinophilic spindle cells arranged in fascicles and sheets and can be distinguished from EBV-SMT by immunoreactivity for follicular dendritic cell markers CD23 and CD35. Like EBV-SMT, FDC sarcoma usually demonstrates immunoreactivity with the EBV receptor CD21. A dural-based EBV-SMT must be distinguished from a meningioma. There are many histologic variants of meningioma, but the most common variants demonstrate whirling, round-to-oval nuclei, some with intranuclear inclusions, eosinophilic cytoplasm and indistinct cytoplasmic borders. Immunohistochemically, meningiomas can be distinguished from EBV-SMT as they are usually positive for EMA, but negative for markers of smooth muscle.

### 3.7. Management and Outcome Data

Even though EBV-SMTs frequently present with multiple lesions, their clinical prognosis appears to be more favorable than conventional LMS. Our review of the literature indicates that even EBV-positive leiomyomas in these AIDs patients have the potential to recur or behave aggressively, and that histologically malignant-appearing tumors may respond well to therapy and demonstrate a relatively favorable outcome. The histologic features of EBV-SMT did not correlate well with the clinical outcome [18, 26]. EBV-SMTs diagnosed as low-grade smooth muscle neoplasms such as leiomyoma appear to progress very slowly, even in the absence of treatment [32, 37]. Compared to conventional LMS that often progresses with hematogenous spread and distant metastasis, EBV-SMT appears to be much less aggressive. Only 35% of patients in these published cases died. Even then, death in these patients was almost four times more likely to be attributed to another condition (e.g., opportunistic infection) and not the patient's SMT. These case reports show that only four patients with EBV-SMT died as a direct consequence of their SMT. Moreover, it does not appear that the patient's age, gender, CD4 cell count, tumor type, tumor size, nor location of their tumor had a noticeable impact on their outcome. There was little difference between tumors that presented within one year and after one year of an HIV diagnosis with respect to the patient age, CD4 count, number of tumors or patient outcome.

Data regarding treatment was available in 36 (56%) patients. Surgical resection was the main therapeutic approach in this published series. Complete remission was achieved in two cases of unifocal EBV-SMT treated with complete surgical excision and HAART [18]. Complete surgical removal for EBV-SMT, however, was usually not possible due to the multifocal nature of this condition. For patients with leiomyomas, three tumors were resected and no therapy was noted for one 5 year old male patient who died 2 months after his diagnosis unrelated to his SMT. In patients diagnosed with LMS, 11 underwent surgical resection, including a splenectomy for one individual [12]. LMS was treated with radiation in three cases, two in combination with resection [14, 16] and one together with chemotherapy (gemcitabine) [7]. EBV-SMT is reported to be resistant to cytotoxic chemotherapy, which may be poorly tolerated by these severely immunocompromised patients [7, 51]. While the impact of antiretroviral therapy could not be evaluated because of limited published data, there are rare published cases in which the patients' tumor remained stable in size or regressed even with a modest CD4 response to highly active antiretroviral therapy (HAART) [4, 32, 42]. This is similar to the treatment of patients with PTLD, where improvement of the immune status improves outcome in patients with EBV-SMT [4, 18]. In one case of LMS, a 7-year-old-girl was treated with antiretrovirals only and was alive with her tumor after 3 years of follow-up [42]. In 15 cases where the SMT was not further subtyped, or called SMT-UMP (2 cases), therapy involved tumor resection alone, except for one patient who had received additional radiation for an incompletely resected dural SMT [24]. 

Yin et al. recently reviewed the literature regarding therapeutic strategies for treating leiomyoma and LMS in children with AIDS. Based on the fact that there are no randomized or clinical controlled trials and most of the data is based on case reports and small series, they conclude that it is likely best to treat each patient with a case-by-case manner until larger, more rigorous studies are carried out [57]. In some cases, conservative management and simple observation alone may be appropriate for some of these extremely ill patients [27, 32]. Newer studies have raised the possibility of using EBV-specific immunotherapy, mTOR inhibitors, and demethylating agents as possible therapeutic options for EBV-SMT [51]. The mTOR pathway plays an important role in regulating cell growth and proliferation and its deregulation is associated with many human cancers and diabetes. Sirolimus inhibits mTOR and may be a potential therapeutic alternative for cancers for which the mTOR-Akt pathway is activated. Some success has been seen treating EBV-SMT and Kaposi sarcoma with sirolimus [51–53].

Information about patient outcome was documented in 47 cases (73%), and is summarized in Table 5. Death in these published cases was three times more likely to be due to another etiology than the patient's SMT for both benign and sarcomatous SMT. There did not appear to be any appreciable difference among the various clinical outcomes with respect to patient age or gender, CD4 cell count, tumor type or size, nor location of tumor.

## 4. Conclusion

This paper offers, to the best of our knowledge, the largest analysis to date on cases of SMT arising in the setting of HIV infection. This study is limited by the inclusion of only English language articles. Also, the data may be biased by the absence of published cases from regions of the world (e.g., Africa) where the AIDS epidemic is rampant. This may explain why the bulk of published cases were from the USA. Nevertheless, analysis of these 53 published articles provides some insight into the pathology and behavior of these unique tumors. Absence of specific data elements and tumor subtype in several articles may have limited some of the analyses. 

There is no convincing evidence of viral integration into the host (smooth muscle) genome. The presence of EBV in SMT suggests a causal involvement of EBV in SMTs in AIDS patients. The mode of EBV entry into smooth muscle cells remains speculative. These data highlight the variable aggressiveness and clinical outcome of EBV-SMT arising in patients with AIDS. EBV-SMT should always be included in the differential diagnosis for a mesenchymal tumor arising in any organ in HIV-infected patients of all ages. Further case reports are anticipated as the HIV epidemic persists and awareness of these distinct tumors grows [58]. As pathologists may not always know the clinical history of immunosuppression, it may be wise to include EBV-SMT in the differential diagnosis of any smooth muscle tumor occurring in uncommon sites. Future work should be directed at determining why certain individuals are susceptible to EBV-mediated transformation. It also remains to be seen what impact HAART has on these unique neoplasms and whether newly described lines of therapy such as mTOR inhibitors and demethylating agents will have beneficial therapeutic effects. Larger studies are needed to improve our understanding and management of these unique EBV-SMTs.

## Figures and Tables

**Table 1:** Countries reporting SMT in patients with HIV/AIDS.

Region	Country	# of Publications	# of SMT cases
North America	USA	19	38
	Canada	1	1

Asia	Thailand	3	11
	Hong Kong	2	3
	Taiwan	1	1
	Singapore	1	1

Europe	France	4	5
	Spain	2	2

South America	Argentina	1	1
	Peru	1	1

**Table 2:** Age and gender distribution of SMTs in patients with HIV/AIDS.

Patient age (years)	Male	Female
<10	7	12
10–20	1	2
>20	28	14

**Total Cases**	**36**	**28**

**Table 3:** Clinicopathological characteristics of benign and malignant EBV-SMT (data from available published cases).

Tumor	Leiomyoma	Leiomyosarcoma
Number of cases	10	25
Patient age average (range) in years	11 (2–36)	20 (5–48)
Male patients	7	8
Female patients	3	17
CD4 cell count mean (cells/*μ*L)	44.5	60
Time to presentation (months)	42.8	57
Tumor size (cm)	0.5–5	1–7
Tumor site	Brain, lung, liver, spleen, adrenal, node, soft tissue, extremity	Brain, spinal cord, lung, liver, spleen, adrenal, gastrointestinal tract, vertebrae, node, soft tissue, extremity, serosa, eye, ethmoid sinus, vulva
Solitary tumor	3	9
Multiple tumors	7	11
Patient fatal outcome	6	8

**Table 4:** Immunohistochemical stains with positive immunoreactivity in EBV-SMT.

Immunostain	Positive cases	Negative cases
Smooth muscleactin (SMA)	39	0
alpha-smooth muscle actin (AMA)	17	0
Muscle-specific actin (MSA)	9	0
Desmin	42	6
Vimentin	5	2
CD21 (C3d Receptor; EBV receptor)	9	2
Caldesmon	1	0
Myosin Smooth muscle heavy chain (SMHC)	1	0
Calponin	1	0

**Table 5:** Outcome of SMT in HIV-infected patients.

Clinical outcome	Number of patients (*N* = 64)	Months recorded average (range)
Alive with no evidence of SMT	8 (13%)	9 (5–20)
Alive with SMT	19 (30%)	17 (0.5–60)
Died of SMT	4 (6%)	7 (4–12)
Died of other cause(s)	14 (22%)	3 (0–11)
Lost to follow up	2 (3%)	Not applicable
No published data available	17 (27%)	Not applicable

LMS: leiomyosarcoma.
